# Tunnelled Peritoneal Catheter for Malignant Ascites—An Open-Label, Prospective, Observational Trial

**DOI:** 10.3390/cancers13122926

**Published:** 2021-06-11

**Authors:** Golo Petzold, Sebastian C. B. Bremer, Felix C. Heuschert, Hannes Treiber, Volker Ellenrieder, Steffen Kunsch, Albrecht Neesse

**Affiliations:** 1 Department of Gastroenterology, Gastrointestinal Oncology and Endocrinology, University Medical Center Goettingen, 37075 Goettingen, Germany; golo.petzold@med.uni-goettingen.de (G.P.); sebastian.bremer@med.uni-goettingen.de (S.C.B.B.); f.heuschert@stud.uni-goettingen.de (F.C.H.); volker.ellenrieder@med.uni-goettingen.de (V.E.); steffen.kunsch@med.uni-goettingen.de (S.K.); 2 Department of Hematology and Oncology, University Medical Center Goettingen, 37075 Goettingen, Germany; hannes.treiber@med.uni-goettingen.de

**Keywords:** malignant ascites, tunnelled peritoneal catheter, PleurX catheter, symptomatic ascites

## Abstract

**Simple Summary:**

Recurrent abdominal fluid collection (ascites) is a common clinical problem in patients with advanced malignancy that causes abdominal discomfort, nausea, fatigue and dyspnoea. Repetitive large volume paracentesis is the standard procedure that relieves patients from abdominal fluid; however, the procedure is painful and needs to be repeated up to several times per week. Tunnelled peritoneal PleurX catheters are implanted in the abdominal cavity as a permanent solution to drain ascites and can be used by the patient or nursing staff at home. In this study, we prospectively investigate the feasibility and safety of tunnelled peritoneal PleurX catheters in patients with malignant ascites. Our findings show that this technique is safe and can be routinely used. Tunnelled peritoneal PleurX catheters effectively reduce ascites-associated symptoms and hospitalization rates. Therefore, this technique should be considered in patients with recurrent malignant ascites.

**Abstract:**

Treatment of recurrent malignant ascites in cancer patients is a challenge. Evidence based guidelines regarding the best treatment strategy are lacking. The aim of this prospective study was to investigate the safety and efficacy of a tunnelled peritoneal catheter (PleurX) in cancer patients with symptomatic ascites. Patients with symptomatic, diuretics-refractory ascites and indication for the implantation of a tunnelled peritoneal PleurX catheter were prospectively enrolled between August 2018 and July 2020. The number of catheter days, complications, amount of drained ascites and ascites-associated symptoms and hospitalization rate pre- and post-PleurX insertion were analysed. 51 Patients (64.7% male) were prospectively enrolled. The mean age was 66.6 (±7.9) years. The most common cause of ascites was pancreatic adenocarcinoma (*n* = 10) followed by cholangiocellular carcinoma (*n* = 9) and hepatocellular carcinoma (*n* = 8). The technical success rate of PleurX implantation was 100%. The mean volume of weakly drained ascites was 5.44l (±4.08). Major complications included cellulitis (*n* = 2), peritonitis and drainage dislocation (each *n* = 1). The mean catheter days per patient was 59.8 (±107.4) (Min 4, Max 668). Abdominal discomfort, impaired mobility, dyspnoea, fatigue, nausea and vomiting were significantly reduced 30 days after PleurX insertion (*p* < 0.05). Moreover, hospitalization rate was significantly reduced (*p* < 0.001; 27.08% of days preimplantation vs. 11.27% postimplantation). We conclude that implantation of a tunnelled ascites catheter is a safe and effective method for the treatment of refractory ascites in cancer patients with advanced disease. Serious complications are rare. Burdensome ascites-associated symptoms and hospitalization rates can be significantly reduced over a longer period of time.

## 1. Introduction

Advanced malignancies with abdominal manifestation are a common cause of ascites. Large amounts of abdominal fluid collections lead to patient discomfort, especially nausea, vomiting, dyspnoea and fatigue. Therefore, the treatment of recurrent malignant ascites in cancer patients is a challenge. Due to the lack of prospective and controlled clinical studies, there is no current oncology guideline that recommends a particular treatment for recurrent malignant ascites. The use of diuretics can be attempted, but in the majority of patients, an adequate reduction of ascites with diuretics alone is not possible [1]. In these cases, the standard therapy is repetitive large volume paracentesis [2], which can lead to a transient reduction of ascites associated symptoms [3]. The disadvantages are the repeated invasive and painful procedures with potential complications, such as infection and bleeding, and the need for in- or outpatient visits. An alternative therapy option is the implantation of an automated low-flow ascites pump system (alfapump). This procedure leads to a reduction in the number of paracenteses and an improvement of quality of life. However, this procedure is rather expensive and associated with a number of complications, such as renal failure, infections and dysfunction of the pump system [4]. The implantation of peritoneovenous shunts is another option, but contraindicated for ascites due to gastrointestinal malignancies. In addition, this procedure is complex and costly [2]. Another possibility to remove ascites in palliative care patients is the implantation of a tunnelled peritoneal catheter. A few small retrospective studies on the safety and efficacy of this procedure exist [5,6,7,8,9,10,11,12,13], but prospective studies are lacking. The aim of our open-label, prospective, observational study was to investigate the safety and efficacy of a tunnelled peritoneal catheter in cancer patients with symptomatic diuretics-refractory ascites with particular focus on the effect on ascites-associated symptoms and the number of days spent in hospital.

## 2. Material and Methods

### 2.1. Inclusion Criteria

Patients with symptomatic, diuretics-refractory ascites and indication for the implantation of a tunnelled peritoneal PleurX catheter were prospectively included between August 2018 and July 2020. Catheter implantation was performed in the Department of Gastroenterology, Gastrointestinal Oncology and Endocrinology at the University Medical Center Goettingen, 37075 Goettingen, Germany.

Patient-related data, laboratory values and ascites-associated symptoms prior to implantation were recorded; technical success rate, complications, overall survival and hospitalization rate were documented and evaluated. A standardized phone interview was conducted one month and three months after catheter insertion to collect data about quality of life, changes in ascites-associated symptoms (using a five-point scale), days of hospitalization and amount of drained ascites volume per week.

In cases of incomplete data collection from the patient, the family doctor or persons authorized by the patient to manage their affairs were contacted and interviewed. Follow up was performed until the death of the patients.

The study was approved by the local institutional ethics committee (Case No. 10/6/18). All patients signed an informed consent form in accordance with the Declaration of Helsinki (2013).

### 2.2. PleurX Peritoneal Catheter

PleurX tunnelled peritoneal drainage catheters (PleurX; CareFusion Corporation, San Diego, CA, USA; local distributor: Fa. Ewimed, Hechingen, Germany) were used in all patients. The PleurX peritoneal drainage catheter is a 15.5 French, 71-cm fenestrated silicone catheter with a one-way valve mechanism and a polyester cuff. After connecting a drainage bag to the exit of the catheter, the patient can independently drain ascites if necessary.

### 2.3. Implantation of the Catheter

**Figure 1:**
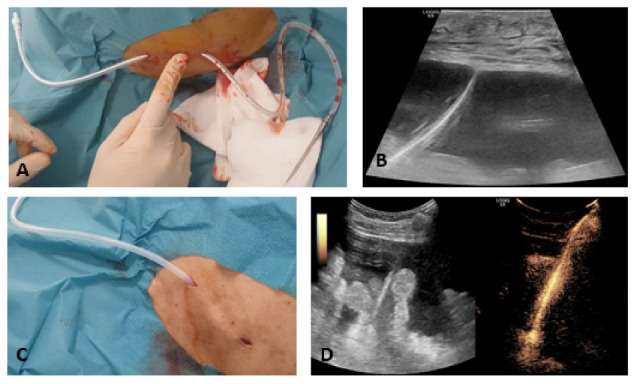
(**A**–**D**): Subcutaneous tunnelling of the PleurX catheter. The fingertip points to the position of the polyester cuff (**A**); sonographic control of guidewire position in the abdominal cavity (**B**); after using the peel-away introducer, the fenestrated part of the catheter is now inside the abdominal cavity (**C**). Optionally, the position of the catheter in the abdominal cavity can be verified by administering a few drops of SonoVue over the drainage (**D**).

Implantation of the catheter was performed in the Unit of Endoscopy and Sonography of our department by experienced physicians after written informed consent. Implantation was performed in local anaesthesia using mevivacaine 1% (Deltamedica, Reutlingen, Germany) and, depending on patients’ and physicians’ choice, additional analgesic sedation using propofol (Baxter, Utrecht, The Netherlands) and/or piritramide (Hameln Pharma plus, Hameln, Germany). Before implantation, an abdominal ultrasound was performed to rule out chambered ascites and to determine the most appropriate position for catheter placement. Catheter implantation was performed according to the manufacturer’s instruction. The guidewire was placed in the abdominal cavity under sonographic control. The length of the subcutaneous tunnel was 5 to 8 cm. The exit of the catheter was placed cranial or caudal to the tunnel, depending on the operator’s choice and the predominant accumulation of ascites. Optionally, the position of the catheter in the abdominal cavity was controlled by administering a few drops of SonoVue (Bracco, Konstanz, Germany) together with saline solution over the drainage (Figure 1A–D). Upon catheter implantation, a single shot antibiotic with 2 g ceftriaxone (Roche Pharma AG, Grenzach-Wyhlen, Germany) was applied intravenously. Technical success was defined as the insertion of the catheter in an appropriate intraperitoneal position with successful ascites drainage.

### 2.4. Statistical Analysis

Statistical analysis was performed using SPSS (Version 26 (IBM, Armonk, NY, USA)) and GraphPad prism, Version 8 (GraphPad, San Diego, CA, USA). We evaluated the normal distribution of quantitative variables. Data were reported as mean including standard deviation. An independent t-test and Mann–Whitney U-test, respectively, were performed to compare differences between two groups. We defined a statistically significant difference as *p* < 0.05. The sign test was used to compare the burden of ascites-associated symptoms before and after implantation of PleurX catheter. We used the Pearson correlation coefficient to measure linear correlation between the number of catheter days and patient-related parameters.

## 3. Results

### 3.1. Patient Characteristics

In total, 51 patients were prospectively enrolled, 33 (64.7%) of them being male. The mean age was 66.6 (±7.9) years, and the mean BMI was 25.46 (±4.1) kg/m^2^. The most common cause of ascites was pancreatic adenocarcinoma (*n* = 10) followed by cholangiocellular carcinoma (*n* = 9) and hepatocellular carcinoma (*n* = 8). Twenty-two patients were under regular antitumor therapy at the time of the drainage implantation; in the remaining 29 patients (56.9%), a “best supportive care” concept was established. Patients’ characteristics are shown in Table 1. Patients underwent a mean of 2.20 (±1.67) large volume paracentesis procedures before the PleurX placement (range 0–7 procedures). Within three months before implantation of the PleurX drainage, ascites was drained using an indwelling peritoneal catheter system (8F or 10F button drainage) for 3 to 10 days in 16 patients (31.4%).

**Figure 2:**
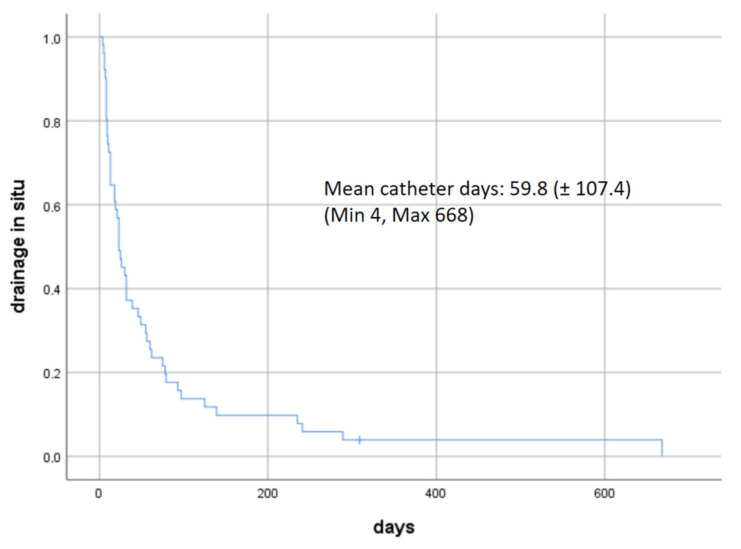
Kaplan–Meier curve (*n* = 51). Time from PleurX insertion to loss of drainage (death (*n* = 46) and explantation (*n* = 5), respectively).

The technical success rate of PleurX catheter implantation was 100%. Mean catheter days per patient was 59.8 (± 107.4) (min 4, max 668 days). Figure 2 shows the time from PleurX insertion to loss of drainage (death (*n* = 46) and explantation (*n* = 5), respectively) using a Kaplan–Meier curve. Thirty days after drainage implantation, the catheter was still in situ in 23 of 51 patients (45.1%). Twenty-seven patients (52.94%) died within 30 days after catheter insertion.

### 3.2. Ascites-Associated Symptoms

All patients had ascites-associated symptoms prior to catheter implantation. The majority of reported symptoms were abdominal discomfort (95.7% of all patients), followed by impaired mobility (87.0%), physical weakness (85.1%), dyspnoea (71.7%), fatigue (68.1%), nausea and vomiting (48.9%). Thirty days after catheter implantation, the symptoms could be assessed in 17 of 23 patients, and the burden of symptoms was significantly reduced (see Table 2). A total of 85.7% of patients reported an improved general condition 30 days after PleurX insertion. Furthermore, our data reveal that symptoms such as dyspnoea and abdominal pressure were markedly reduced three months after implantation of the catheter (each *p* = 0.063).

The mean volume of weekly drained ascites was 5.44l (±4.08). Three months after implantation (five patients were still alive), the amount of ascites remained stable (5.18l (±5.20). In most cases, ascites was drained by home nursing care services (40.0%), followed by the patient him/herself (33.3%) or by relatives (27.6%) after appropriate instruction by the company Ewimed.

### 3.3. Catheter-Associated Complications

Major complications were cellulitis (*n* = 2), peritonitis and drainage dislocation (each *n* = 1). In all cases, the catheter had to be explanted. Overall, explantations were carried out in five cases due to cellulitis (at days 13 and 668, respectively), peritonitis (at day 39), dislocation (at day 13) or absence of ascites (at day 289). In two cases, re-implantation was performed after antibiotic therapy. Reported minor complications were erythema next to the catheter insertion site (*n* = 8), leakage of ascites (*n* = 5), occlusion of the catheter (*n* = 3), pain during the drainage of the ascites (*n* = 2) and hematoma (*n* = 1). Occlusion of the catheter could be resolved in all cases after rinsing with saline. All other minor complications were only transient and did not require special treatment.

### 3.4. Hospitalization

**Figure 3:**
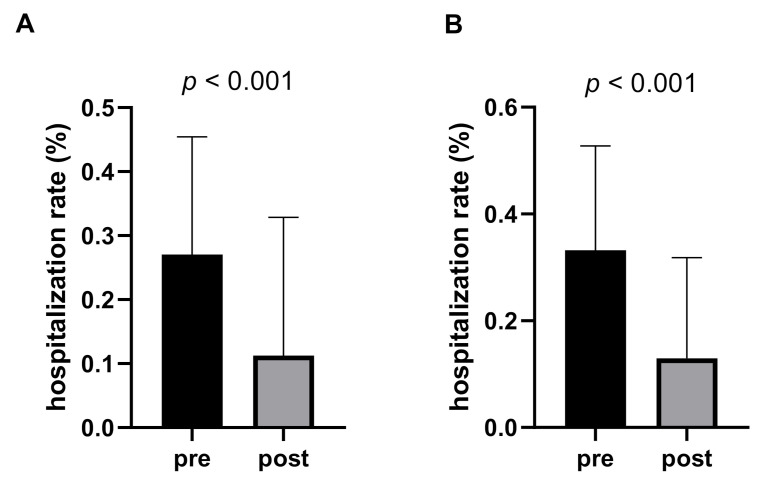
Hospitalization rate (% days) within 90 days pre-PleurX insertion (“pre“) and within 30 days post discharge from the hospital (“post“). (**A**): all patients (*n* = 51); (**B**): patients with drainage in situ after 30 days (*n* = 23).

Three months before catheter implantation, the mean duration of days in the hospital was 24.37 (±16.53) days. Upon catheter implantation and discharge from the hospital, mean hospitalization rate within the next 30 days and until death, respectively, was 11.27% (±21.59) compared to 27.08% (±18.36) before implantation (*p* < 0.001, Figure 3A). Twenty-four patients (47.1%) did not have to be readmitted after discharge from the hospital and died on average after 25.8 days at home or in hospice.

The mean time period between catheter implantation and discharge from the hospital was 5.69 (±5.99) days.

Within the subgroup of patients that could be assessed 30 days after catheter implantation (*n* = 23), the rate of hospitalization was 33.24% (±19.56) three months before catheter implantation compared to 12.95% (±18.90) 30 days after implantation (*p* < 0.001, Figure 3B). Within the subgroup of patients that could be assessed 3 months after catheter implantation (*n* = 9), the rate of hospitalization was 6.94 % (±9.33) compared to 39.63 % (±20.83%) 3 months before implantation (*p* < 0.001). Thus, independent of the duration of the catheter use (> 30 days or > 90 days), the rate of hospitalizations was significantly reduced.

### 3.5. Predictors of Catheter Days Per Patient

Performing correlation analysis, the parameters LDH (ascites) (r = −0.035), CRP (serum) (r = −0.191), leucocytes (ascites) (r = 0.117) and age (r = −0.060) were not significantly correlated with the duration of the catheter per patient. However, treatment status (“antitumor therapy” versus “best supportive care”) (r = 0.348) was significantly correlated (*p* = 0.012) with the duration of the catheter per patient. In patients receiving antitumor therapy, the number of catheter days was significantly higher (108.3 (±151.5) than in patients with the “best supportive care” concept (31.0 (±47.0); *p* < 0.001).

## 4. Discussion

Recurrent malignant ascites is a frequent clinical dilemma in patients with advanced malignancies. To date, no standardized evidenced-based guidelines exist for the management of these patients, and prospective data are lacking. In our open-label, prospective, observational trial, the technical success rate of catheter placement was 100%. This is in line with published data [14]. The implantation was also safe in seriously ill patients with a very short life expectancy, and there was no procedural death. The rate of complications was low, and major complications such as cellulitis, peritonitis and catheter dislocation occurred between 1 and 4% of cases. Minor complications included transient erythema, leakage of ascites and occlusion. Complications such as catheter dysfunction, leakage of ascites and catheter dislocation are reported with a rate of up to 20% in the literature [14]. Interestingly, our results show that no additional paracenteses were necessary upon implantation of the catheter.

All patients suffered from ascites-associated symptoms prior to catheter implantation. More than 80% of patients reported an improved general condition 30 days after PleurX insertion. Improvement also remained 3 months after implantation (six of six patients reported a better general condition). Thirty days after PleurX insertion, ascites-associated symptoms were significantly reduced, especially nausea and vomiting, abdominal discomfort, dyspnoea, fatigue and impaired mobility. Three months after catheter implantation, symptom relief persisted. Notably, these aspects were not investigated in previous studies. Only the study by Courtney and colleagues that included 34 patients focused on ascites-associated symptoms. In this cohort, 2 and 8 weeks after catheter implantation, the symptoms “feeling bloated”, abdominal discomfort, diarrhoea and nausea were significantly reduced. However, 56% of patients rated their overall quality of life as improved in the first week after catheter placement, but at week 12, only 28% (two of seven) stated that their overall quality of life had improved [14].

In our study, hospitalization rate after PleurX insertion was significantly lower than three months before catheter implantation. In addition, the majority of seriously ill patients with a very short life expectancy (≤1 month) did not have to be rehospitalized after catheter implantation following discharge. There is only one prior study that examined the hospitalization rate pre- and post-catheter implantation [9]. The authors showed a mean difference of 4.2 hospital days per month (10.0 pre-catheter vs. 5.8 post-catheter). In our study, we recorded the hospitalization rate three months before catheter implantation. Calculated for one month before and after implantation, the difference was 4.9 days and thus comparable to the study mentioned.

Alternatively, a percutaneous peritoneovenous (Denver) shunt has been reported for the treatment of recurrent malignant ascites. However, most case studies and small retrospective trials reported a relatively high complication rate of up to 40% including severe disseminated intravascular coagulation (DIC), volume overload, shunt occlusion and potential hematologic dissemination [2,15,16]. Even though a direct randomized comparison would be required to draw final conclusions, our current data suggest that implantation of a tunnelled PleurX peritoneal catheter is superior and less invasive than Denver shunts in severely ill cancer patients.

In summary, we can conclude that the implantation of PleurX peritoneal catheter in cancer patients with symptomatic ascites leads to a marked reduction of ascites-associated symptoms in combination with an improved general condition. Importantly, the rate of hospitalization was significantly reduced upon PleurX implantation.

The number of catheter days and the survival time of the patients in our collective was very variable, possibly due to a highly heterogeneous study population, different tumour entities and treatment algorithms. The performance status (fit for tumour therapy) was the decisive positive predictor for the number of catheter days. Interestingly, CRP blood value, LDH value and number of leucocytes in ascitic fluid had no impact on catheter days and on catheter-associated complications.

The main alternative to the tunnelled peritoneal catheter is repetitive large volume paracentesis. Bohn and Ray examined the time point at which tunnelled peritoneal catheter placement becomes less costly than repeat large-volume paracentesis (LVP) for patients with malignant ascites. The authors concluded that use of a tunnelled peritoneal catheter improves the cost advantage for patients who receive LVP more frequently or patients who have less than 5 L of fluid drained per procedure [17]. Another alternative to LVP or a tunnelled peritoneal catheter is the implantation of an automated low-flow ascites pump system (alfapump). While this therapy option is even mentioned in national guidelines for patients with ascites due to liver cirrhosis [18], data quality on its use in malignant ascites is low. Reported complication rates with the alfapump appear to be higher in comparison to PleurX drainage. The most common complications are pump or catheter dysfunction, acute kidney failure and infections (each 23.5%) [19]. Notably, a few patients required additional paracentesis, despite an implanted alfapump, which was not necessary for tunnelled PleurX catheters.

Potential limitations of our study include the use of a single-group design and the lack of laboratory results after catheter implantation. In particular, a control group with a simple catheter (non-tunnelled) for 3–5 days and simple drainage for patients that died within the first 30 days upon implantation of the tunnelled catheter would have been helpful to further tailor this therapeutic option to the most appropriate patient cohort. Additionally, data collection post catheter insertion was exclusively performed by telephone interview and not by direct doctor-patient contact. Thus, we cannot exclude a reporting bias, in particular for the amount of weekly drained ascites. Furthermore, no information regarding ascites-related complications could be collected in patients that died before 30 days after catheter implantation.

## 5. Conclusions

This is the first prospective and long-term observational study that investigated the effect of tunnelled PleurX catheters on ascites-associated symptoms and hospitalization rates. Implantation of a peritoneal PleurX catheter is a safe and effective method for the treatment of refractory ascites in palliative patients with advanced tumour disease. Serious complications are rare. Burdensome ascites-associated symptoms and hospitalization rates can be significantly reduced over a longer period of time. Therefore, we recommend early consideration of the implantation of tunnelled peritoneal catheters in patients with recurrent ascites.

## Figures and Tables

**Table 1:** Characteristics of the patients. CRP: C-reactive protein, LDH: lactate dehydrogenase; BMI: body mass index.

Characteristic	*n* = 51
Female (*n*)	18 (35.3%)
Male (*n*)	33 (64.7%)
Age (years)	66.6 (±7.9)
Body mass index (BMI) (kg/m^2^)	25.46 (±4.1)
Malignant disease (*n*)	51 (100%)
Pancreatic adenocarcinoma	10 (19.6%)
Cholangiocellular carcinoma	9 (17.6%)
Hepatocellular carcinoma	8 (15.7%)
Gastric cancer	5 (9.8%)
Colorectal cancer	3 (5.9%)
Ovarian cancer	3 (5.9%)
Others	13 (25.5%)
Under treatment (*n*)	22 (43.1%)
Best supportive care (*n*)	29 (56.9%)
CRP (mg/dl)	98.5 (±79.5)
LDH ascites (U/I)	345.0 (±40.4.2)
Leucocytes ascites (10^3^/µL)	0.55 (±1.07)
Hospitalization rate 90 days pre implantation (days)	24.86 (±14.32) (27.08 (±18.36)%)

**Table 2:** Symptoms 30 days and 3 months after implantation of PleurX catheter compared to the time before implantation. “+” = decrease in symptoms in comparison to the time before PleurX insertion; “=“ = no change in symptoms; “− “ = increase in symptoms; “change” = mean change of symptoms after PleurX insertion using a five point scale (−2 = significant deterioration; −1 = slight deterioration; 0 = no change; 1 = slight improvement; 2 = significant improvement).

	After 30 Days (*n* = 17)					After 3 Month (*n* = 6)				
**Symptom**	+	=	−	change	*p*	+	=	−	change	*p*
dyspnoea	10	6	0	1.19	0.02	5	1	0	1.50	0.063
abdominal discomfort	14	2	0	1.38	<0.001	5	0	0	1.40	0.063
fatigue	6	10	0	0.62	0.031	2	4	0	0.67	0.5
nausea/vomiting	9	6	1	1.13	0.021	3	3	0	0.83	0.25
physical weakness	5	9	1	0.40	0.219	2	2	2	0.17	1
impaired mobility	11	6	0	1.00	<0.001	4	2	0	1.00	0.125

## Data Availability

De-identified individual data might be made available following publication by reasonable request from the corresponding author.
